# High-Dose Methotrexate Usage Without Drug-Level Monitoring in Advanced Pediatric Mature B-Cell Non-Hodgkin Lymphoma in a Resource-Limited Setting in Malawi

**DOI:** 10.1200/GO-24-00591

**Published:** 2025-03-28

**Authors:** Rizine R. Mzikamanda, Loviisa Mulanje, Casey L. McAtee, Apatsa Matatiyo, Zoe Mwale, Grace Chirwa, Watipaso Wanda, Atupele Miranda Mpasa, Stella Wachepa, Minke H.W. Huibers, Steve Martin, Tamiwe Tomoka, Maurice Mulenga, Yuri Fedoriw, Gugulethu Mapurisa, Julie M. Gastier Foster, Nader El-Mallawany, Katherine D. Westmoreland, Peter Wasswa, Carl E. Allen, Nmazuo Ozuah

**Affiliations:** ^1^ Pediatric Hematology-Oncology, Kamuzu Central Hospital, Lilongwe, Malawi; ^2^ Baylor College of Medicine Children's Foundation—Malawi, Lilongwe, Malawi; ^3^ Department of Epidemiological Methods and Etiological Research, Leibniz Institute of Prevention Research and Epidemiology, Bremen, Germany; ^4^ Department of Pediatrics, Baylor College of Medicine, Houston, TX; ^5^ Global HOPE, Texas Children's Hospital, Houston, TX; ^6^ University of North Carolina Project Malawi, Lilongwe, Malawi; ^7^ Department of Pediatrics and Child Health, Kamuzu Central Hospital, Lilongwe, Malawi; ^8^ Princes' Maxima Center Pediatric Oncology, Utrecht, the Netherlands; ^9^ Global Child Health Group, Amsterdam UMC, Amsterdam, the Netherlands; ^10^ Department of Pediatrics, University of South Carolina School of Medicine, Greenville, SC; ^11^ University of North Carolina at Chapel Hill, Chapel Hill, NC; ^12^ Pediatric Hematology-Oncology, University of North Carolina at Chapel Hill, Chapel Hill, NC

## Abstract

**PURPOSE:**

Excellent survival for advanced (stages II with high lactate dehydrogenase, III, and IV) pediatric mature B-cell non-Hodgkin lymphoma (MB-NHL) has been achieved with intensive regimens, but adoption in sub-Saharan Africa is limited by inadequate supportive care. We provide real-world data on treating advanced MB-NHL with high-dose methotrexate (HD-MTX; ≥1,000 mg/m^2^/cycle) where real-time serum MTX monitoring is unavailable.

**METHODS:**

We identified two cohorts—a retrospective (January 2017-December 2020) cohort treated with 1,000 or 3,000 mg/m^2^/cycle of HD-MTX and a prospective (July 2022-July 2023) cohort—with a modified LMB96 protocol containing 3,000 mg/m^2^/cycle of HD-MTX. All doses of HD-MTX were given over 3 hours. Estimates of 12-month event-free survival (EFS) and overall survival (OS) were calculated with abandonment as an event. Clinical toxicity data were available for the prospective cohort.

**RESULTS:**

The retrospective cohort had 108 patients who received HD-MTX 1,000 mg/m^2^ (n = 98, 91%) or 3,000 mg/m^2^ per cycle. The 12-month EFS and OS were 39% (95% CI, 30 to 50) and 54% (95% CI, 44 to 64), respectively. HD-MTX at 3,000 mg/m^2^ had superior EFS: 69% (95% CI, 49 to 96) versus 33% (95% CI, 24 to 46), *P* = .004. The prospective cohort had 38 patients. Two ≥grade 3 mucositis, one acute kidney injury, and three treatment-related deaths (8%) occurred. Seven (18%) abandoned treatment. With a median follow-up of 14.5 months, 12-month EFS and OS were 45% (95% CI, 32 to 65) and 59% (95% CI, 45 to 79), respectively. Most relapses were stage IV: EFS 20% versus 51% (non–stage IV; *P* = .057). Severe malnutrition was associated with OS of 33% versus 58% (normal) or 76% (moderate; *P* = .055).

**CONCLUSION:**

HD-MTX dosed at 3,000 mg/m^2^/cycle is feasible in low-resource settings where routine MTX monitoring is unavailable. Stage IV disease and severe malnutrition may contribute to poorer outcomes.

## INTRODUCTION

Burkitt lymphoma, a common pediatric cancer in sub-Saharan Africa (SSA), represents up to 90% of pediatric mature B-cell non-Hodgkin lymphoma (MB-NHL) in the region.^1-3^ MB-NHL accounts for >30% of patients with childhood cancer in Malawi, with >80% presenting with advanced disease.^2,4,5^

**CONTEXT**

**Key Objective**
Is it feasible, safe, and effective to administer intensive regimens containing high-dose methotrexate (HD-MTX) to treat advanced pediatric mature B-cell non-Hodgkin lymphoma in low-resource settings where routine MTX monitoring is unavailable?
**Knowledge Generated**
Patients who received MTX at 3,000 mg/m^2^/cycle had an event-free survival (EFS) of 69%, significantly higher than the 33% EFS observed in those receiving 1,000 mg/m^2^/cycle. Despite the absence of real-time serum MTX monitoring, locally adapted supportive care measures resulted in relatively low rates of treatment-related toxicities and mortality.
**Relevance**
As treatment centers in low-resource settings build capacity for monitoring MTX serum levels, administering HD-MTX alongside rigorous supportive care, emerges as a viable alternative, potentially leading to improved outcomes in patients with advanced pediatric mature B-cell non-Hodgkin lymphoma.

In high-income countries (HICs), excellent survival has been achieved with intensive regimens containing high-dose methotrexate (HD-MTX), high-dose cytarabine, and rituximab,^6^ coupled with standardized supportive care. Adoption of these intensive regimens in low-resource settings is limited by existing resources and often necessitates modifications to dosing and frequent practice of deriving therapy from adult cyclophosphamide, doxorubicin, vincristine, prednisone-based regimens.^3,6,7^

In SSA, survival for MB-NHL (30%-50%) has not changed since the 1970s. Treatment was initially with cyclophosphamide monotherapy, and augmentation with multiagent chemotherapy has been only modestly effective. Survival rates remain lower than reported in HICs with dose-reduced chemotherapy agents.^3,7-14^

Reiter et al^15^ compared the BFM86 and BFM90 protocols, revealing a crucial role for HD-MTX in improving outcomes for advanced MB-NHL.^3^ At Kamuzu Central Hospital (KCH) in Lilongwe, Malawi, where routine serum MTX monitoring is unavailable, HD-MTX regimens with doses of 1,000-3,000 mg/m^2^/cycle were introduced in 2017. We add to the growing experience of incorporating intensive chemotherapy regimens for pediatric MB-NHL in SSA^16^ by providing real-world data on treating children with HD-MTX (≥1,000 mg/m^2^/cycle) in combination with anthracyclines in a low-resource unit in Malawi, a setting typical of pediatric cancer units throughout SSA.

## METHODS

### Study Design and Setting

KCH, as a referral tertiary center, serves a population of approximately 9 million people from central and northern Malawi. The 30-bed pediatric oncology unit is supported through a collaborative partnership between the Ministry of Health of Malawi, the Texas Children's Global Hematology-Oncology-Pediatric-Excellence Program, and the University of North Carolina Project-Malawi. Through this collaboration, essential resources such as chemotherapy, pathology services, oncology nursing, nutritionists, palliative care, pediatric hematologist-oncologists, and so on have become generally adequate. Nonetheless, limitations, such as those in pediatric intensive care and blood product availability, remain evident. The unit is best described as a level 2 center according to the International Society of Pediatric Oncology framework for adapted therapy guidelines.^17^

### Patients

**FIG 1:**
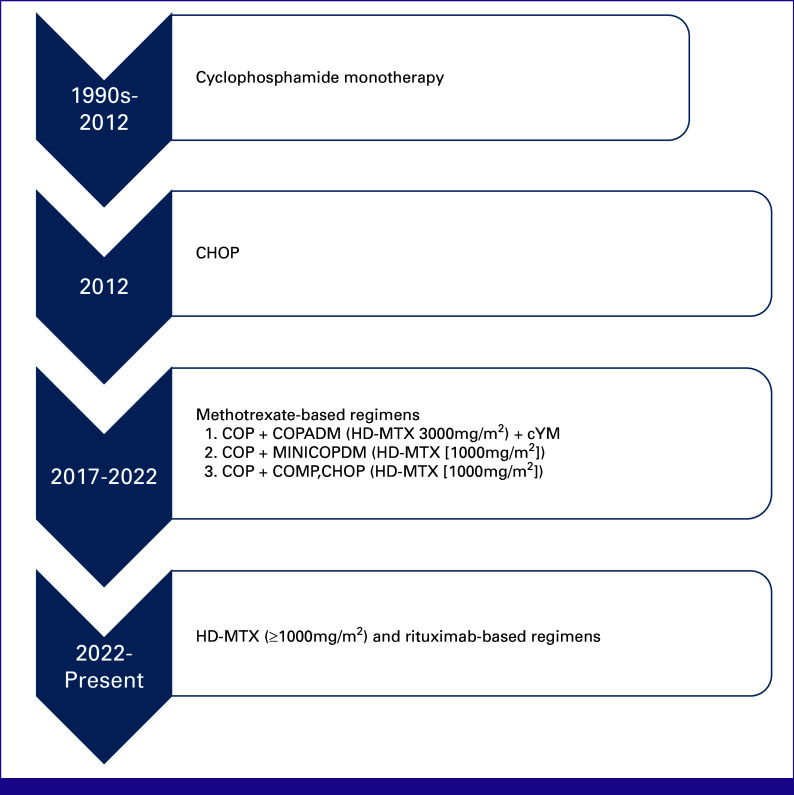
Evolution of chemotherapy regimens for treatment of mature B-cell non-Hodgkin lymphoma at Kamuzu Central Hospital in Malawi from the 1990s till date. The specific regimens and doses of MTX and anthracyclines were administered on the basis of practice patterns at different times. The gradual escalation of chemotherapy regimens and doses is the result of the development of infrastructure to provide improved supportive care. C, cyclophosphamide; CHOP, cyclophosphamide, doxorubicin, vincristine, prednisone; COPADM, cyclophosphamide, vincristine (Oncovin), prednisone, doxorubicin (Adriamycin), HD-MTX (3,000 mg/m^2^/cycle); cY, cytarabine; H/D, doxorubicin hydrochloride (hydroxydaunomycin; 25 or 50 mg/m^2^/cycle based on physician preference); HD-MTX, high-dose methotrexate; M, methotrexate; MiniCOPADM, cyclophosphamide, oncovin, prednisone, Adriamycin (doxorubicin), lower dose MTX (1,000 mg/m^2^/cycle); O, vincristine (Oncovin); P, prednisone.

We identified two cohorts of patients. The first one was a retrospective cohort of patients aged 16 years and younger with advanced MB-NHL treated between January 2017 and December 2020. Chemotherapy regimens and supportive care guidelines were nonstandardized and based on physician preference (Fig 1). Second was a prospective cohort with newly diagnosed MB-NHL treated from July 2022 to July 2023, with a standard of care modified from the FAB/LMB96 protocol,^18^ with addition of rituximab for stage IV, and standardized supportive care guidelines (Table 1; Appendix Table A1).

**TABLE 1:** Treatment Schema for Pediatric MB-NHL at KCH, Lilongwe, Malawi (June 2022-July 2023)

Risk Strata	Phenotype	Chemotherapy Regimen
Standard risk	Stage I/II with low LDH	COP prephase followed by two cycles of COPADM and two cycles of CYM; MTX dose = 1,000 mg/m^2^
High risk	Stage I/II with high LDH stage III regardless of serum LDH amount and non-CNS stage IV disease with BM involvement of <25%	COP prephase followed by two cycles of COPADM and two cycles of CYM; MTX dose = 3,000 mg/m^2^
Very high risk	Stage IV with BM involvement of >25% Stage IV with CNS-positive disease	COP prephase followed by two cycles of R-COPADM, two cycles of R-CYM, and two cycles of maintenance COPADM; MTX dose = 3,000 mg/m^2^

Abbreviations: BM, bone marrow; COP prephase, cyclophosphamide (300 mg/m^2^ once per cycle), vincristine (1 mg/m^2^ once per cycle), prednisone (30 mg/m^2^ twice daily for 7 days) with intrathecal methotrexate, cytarabine, and hydrocortisone; COPADM, cyclophosphamide (1,200 mg/m^2^ once per cycle), vincristine (2 mg/m^2^ once per cycle), prednisone (30 mg/m^2^ twice daily for 5 days per cycle), doxorubicin (25 mg/m^2^ once per cycle), methotrexate (dose based on risk strata) with two intrathecal methotrexate, cytarabine, and hydrocortisone; CYM, cytarabine (100 mg/m^2^ daily for 5 days), methotrexate (dose based on risk strata) with two intrathecal methotrexate, cytarabine, and hydrocortisone; high LDH, a level greater than or equal to two times the ULN; KCH, Kamuzu Central Hospital; LDH, lactate dehydrogenase; low LDH, a level less than two times the ULN; maintenance COPADM, cyclophosphamide (1,000 mg/m^2^ once per cycle), vincristine (2 mg/m^2^ once per cycle), prednisone (30 mg/m^2^ twice daily for 5 days per cycle), doxorubicin (25 mg/m^2^ once per cycle), methotrexate (3,000 mg/m^2^ once per cycle) with two intrathecal methotrexate, cytarabine, and hydrocortisone; MB-NHL, mature B-cell non-Hodgkin lymphoma; MTX, methotrexate; R-COPADM, rituximab (375 mg/m^2^ once per cycle), cyclophosphamide (1,200 mg/m^2^ once per cycle), vincristine (2 mg/m^2^ once per cycle), prednisone (30 mg/m^2^ twice daily for 5 days per cycle), doxorubicin (25 mg/m^2^ once per cycle), methotrexate (dose based on risk strata) with two intrathecal methotrexate, cytarabine, and hydrocortisone; R-CYM, rituximab (375 mg/m^2^ once per cycle), cytarabine (100 mg/m^2^ daily for 5 days), methotrexate (dose based on risk strata) with two intrathecal methotrexate, cytarabine, and hydrocortisone; ULN, upper limit of normal.

All patients were staged according to the Murphy staging system and classified as advanced if they had stage II disease with high lactate dehydrogenase (>two times the upper limit of normal [ULN]), stage III, or stage IV. Staging included clinical assessment and imaging with abdominal ultrasound or computed tomography scans. CNS disease was defined as the presence of lymphoma cells in cerebrospinal fluid (CSF), cranial nerve palsy unrelated to a facial tumor, and clinical signs of an intracranial mass. Routine CSF analysis was unavailable in some patients in the retrospective cohort. Bone marrow (BM) disease was defined as BM infiltration with ≥5% involvement. Patients with 5%-25% infiltration were considered to have BM involvement, and those ≥25% were considered to have Burkitt leukemia (Table 1).

All patients had pathologically confirmed MB-NHL diagnoses based on evaluation of hematoxylin and eosin–stained tissue sections by light microscopy and immunophenotyping by limited immunohistochemistry panel including CD20 and Ki67, and/or flow cytometry. Patient cases were discussed at multi-institutional pathology conference meetings, as previously described (PubMed Identifier: 27594430).^19^ Multiparametric flow cytometry was available in parts of the earlier study period and became more consistently available in the prospective cohort, with diagnosis reliant on demonstration of κ or λ immunoglobin light-chain restriction, CD10 and CD20 expression, and lack of CD34 or cytoplasmic terminal deoxynucleotidyl transferas.^20^ A trained physician or nutritionist routinely assessed all patients at diagnosis and defined it into normal, moderate acute malnutrition (MAM), and severe acute malnutrition (SAM) according to WHO nutrition guidelines.^21^

Retrospective data were extracted from paper charts. Prospective data were collected on case report forms and reviewed weekly. The study received ethical approvals from the institutional review boards at Baylor College of Medicine (Houston, TX) and the National Health Science Research Committee in Malawi. An informed consent was obtained from all patients in the prospective cohort.

### Treatment Received

In the retrospective cohort, HD-MTX regimens were used in combination with other chemotherapy drugs—cyclophosphamide (1,200 mg/m^2^/once per cycle), doxorubicin (25 mg/m^2^/once per cycle or 50 mg/m^2^/once per cycle), vincristine (2 mg/m^2^/once per cycle), cytarabine (100 mg/m^2^/once daily for 5 days/cycle), and prednisone (30 mg/m^2^ twice daily for 5 days/cycle). The specific regimens and doses of MTX and anthracyclines were administered on the basis of practice patterns at different times (Fig 1). In this cohort, 3,000 mg/m^2^/once per cycle of MTX was administered from January to June 2017 and then transitioned to 1,000 mg/m^2^/once per cycle for safety concerns. All MTX doses were administered as 3-hour infusions.

In the prospective cohort, patients were treated with a risk-adapted standard of care containing 3,000 mg/m^2^/cycle of MTX (Table 1), adapted from the group B arm of FAB/LMB96 backbone with a 60% dose reduction of anthracycline.^18^ Rituximab (375 mg/m^2^/once per cycle) and two maintenance cycles were incorporated into the very high-risk (VHR) stratum, defined as patients with any CNS involvement or Burkitt leukemia (BM involvement ≥25%). Treatments were administered in 14-day cycles. Patients not in complete remission (CR) after the first consolidation cycle received four cycles of dexamethasone, cisplatin, high-dose cytarabine, and prednisone. A standardized supportive care guideline for folinic acid and hydration with bicarbonate-containing fluid, which recommends adjustments based on serum creatinine levels in lieu of serum MTX monitoring, was established (Appendix Table A1). Cycles required absolute neutrophils ≥1,000/µL and platelets ≥100,000/µL; normal baseline serum creatinine for age; total bilirubin <1.5 times ULN; and alanine/aspartate transferase <2.5 times ULN. Intravenous (IV) Ringer lactate hydration with 25 mEq/L sodium bicarbonate (NaHCO_3_) at 125 mL/m^2^/h was started 12 hours before MTX and continued for 96 hours. Urine dipstick was obtained twice daily, and if urine pH <7, NaHCO_3_ was increased to 50 mEq/L. Folinic acid rescue started 24 hours after MTX, at 15 mg/m^2^ every 6 hours for 72 hours. Creatinine was checked daily and if >25% the pre-MTX level, fluid and folinic acid were increased to 200 mL/m^2^/h and every 3 hours, respectively, and continued until return to pre-MTX levels; if >50%, folinic acid was increased to 100 mg/m^2^ every 3 hours. In the event of decreased urine output, IV fluid was also increased until output improved.

### Outcomes

In the retrospective cohort, the primary outcomes were 12-month overall survival (OS) and event-free survival (EFS) with 1,000 or 3,000 mg/m^2^ MTX dose regimens. The OS was determined by vital status at the time of right censoring and defined as time between the date of diagnosis and death from any cause. EFS was estimated from the time of diagnosis to the earliest of death, relapse, or treatment abandonment. Treatment abandonment was defined as unplanned absence from curative-intent treatment for ≥28 days.^22^ Patients were right censored on the earlier of the last follow-up date or March 2022. Death was categorized into treatment-related, disease-related, and unknown causes.^23^ Treatment-related death was defined as death not directly related to cancer, occurring in the absence of progressive disease, from treatment-related events, such as febrile neutropenia, and severe sepsis.^24,25^

In the prospective cohort, the primary outcome was frequency of treatment-related adverse events (AEs) as defined by Common Terminology Criteria for Adverse Events version 5.0 criteria, including febrile neutropenia, mucositis, and acute kidney injury (AKI).^26^ The 12-month OS and EFS were also assessed. Patients were right censored on the earliest of the last follow-up date or March 2024. Descriptive statistics were used to report on the frequency of severe AEs.

### Statistical Analysis

Pearson or Fisher chi-squared test was used for comparison of categorical data and Welch *t*-test for continuous data. Kaplan-Meier survival curves and multivariable Cox proportional hazards models were used to identify risk factors associated with survival. Median follow-up time was calculated using the reverse Kaplan-Meier method. All *P* values were two-sided, and a value <.05 was considered statistically significant. All analyses were conducted using R. Data were collected from November 2021 to July 2023, with analysis through September 2024, following Strengthening the Reporting of Observational Studies in Epidemiology guidelines.^27^

## RESULTS

### Baseline Characteristics and Treatment in the Retrospective Cohort

Between January 2017 and December 2020, 208 patients were diagnosed with MB-NHL, among whom 138 (66%) had advanced disease. Our analysis focused on the subset of 108 patients with advanced disease who received HD-MTX. Sixty-one (56%) were male, and the median age was 9 years (IQR, 6-11). Twenty-nine (27%) were stage IV. Seven (13%) had CNS disease; however, CSF analysis was not routine, with only 55 (52%) having documented CSF status (Table 2).

**TABLE 2:** Characteristics of the Pediatric Patients With Advanced MB-NHL Within the Retrospective and Prospective Cohorts at Kamuzu Central Hospital, Lilongwe, Malawi

Patient Characteristic	Retrospective Cohort (n = 108)	Prospective Cohort (n = 38)	*P*
Age, years, median (IQR)	9 (6-11)	8 (6-11)	1.0
Male sex, No. (%)	61 (56)	26 (68)	.27
Nutritional state at diagnosis, No. (%)			.02
Normal	40 (37)	15 (39)	
Moderate acute malnutrition	19 (18)	14 (37)	
Severe acute malnutrition	49 (45)	9 (24)	
Murphy stage at diagnosis,^a^ No. (%)			.25
Stage III	79 (73)	20 (53)	
Stage IV	29 (27)	13 (34)	
CSF positive at diagnosis,^b^ No. (%)	7 (13)	4 (11)	1.0
BMA disease, No. (%)	24 (22)	11 (29)	.54

NOTE. Age between each cohort was assessed with the Welch *t*-test. Differences in proportions were assessed with the Pearson *c*^2^ test.

Abbreviations: BMA, bone marrow aspiration; CSF, cerebrospinal fluid; LDH, lactate dehydrogenase.

^a^ Five patients in the prospective cohort were stage II with high LDH.

^b^ CSF status was missing for 53 (49%) in the retrospective cohort. Proportion here is percentage of those with CSF results (n = 55).

Most patients received chemotherapy combinations with MTX dosed at 1,000 mg/m^2^ (91%) compared with 3,000 mg/m^2^/cycle (9%). In the 1,000 mg/m^2^ group, 28% (27/98) were stage IV, compared with the 3,000 mg/m^2^ group (2/10, 20%; *P* = .27); 36% (35/98) had normal nutrition and 46% with SAM, versus 50% (5/10) with normal nutrition and 40% (4/10) with SAM in the 3,000 mg/m^2^ group (*P* = .06). Both groups did not differ with respect to age, sex, stage, or CNS status (Appendix Table A2).

### Survival Outcomes in the Retrospective Cohort

#### Survival Estimates

**FIG 2:**
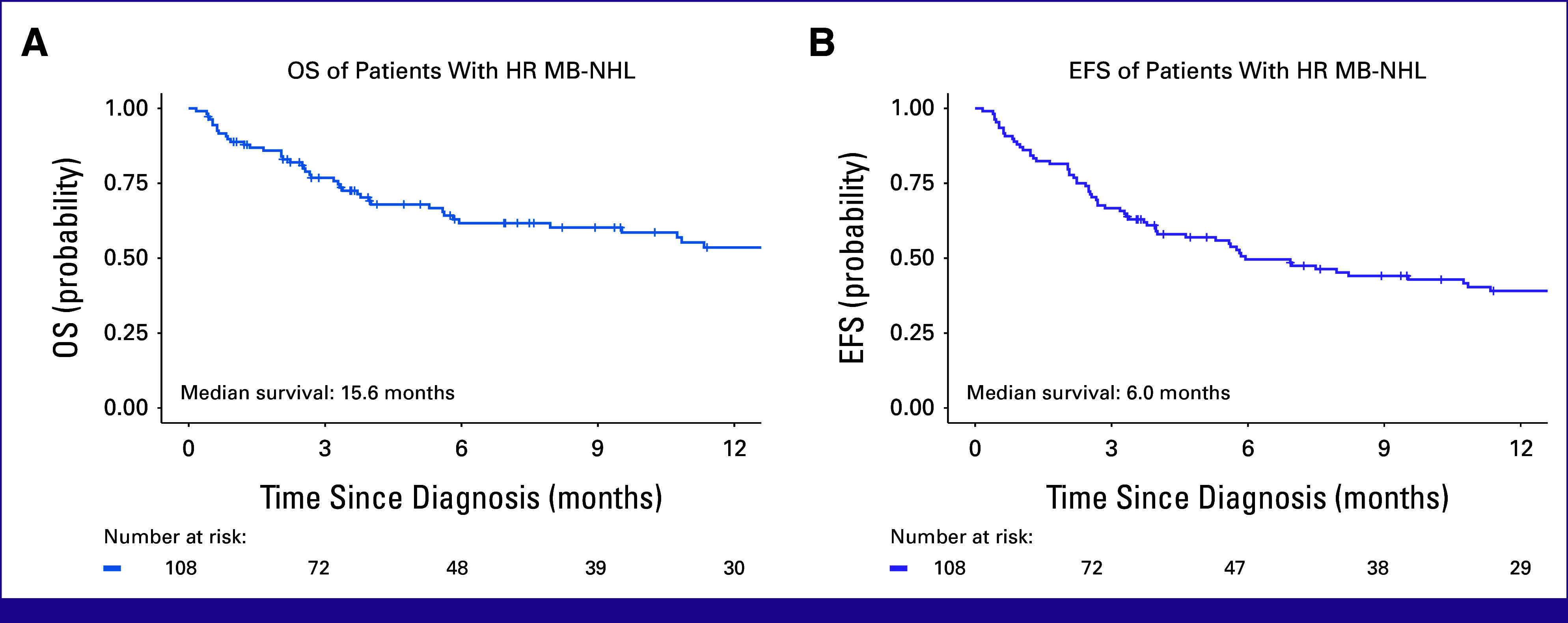
Kaplan-Meier 12-month OS and EFS estimates for a retrospective cohort of children with advanced MB-NHL at Kamuzu Central Hospital in Malawi. The 12-month OS was 54% (95% CI, 44 to 66), and the 12-month EFS was 39% (95% CI, 30 to 50). EFS, event-free survival; HR, high-risk; MB-NHL, mature B-cell non-Hodgkin lymphoma; OS, overall survival.

**FIG 3:**
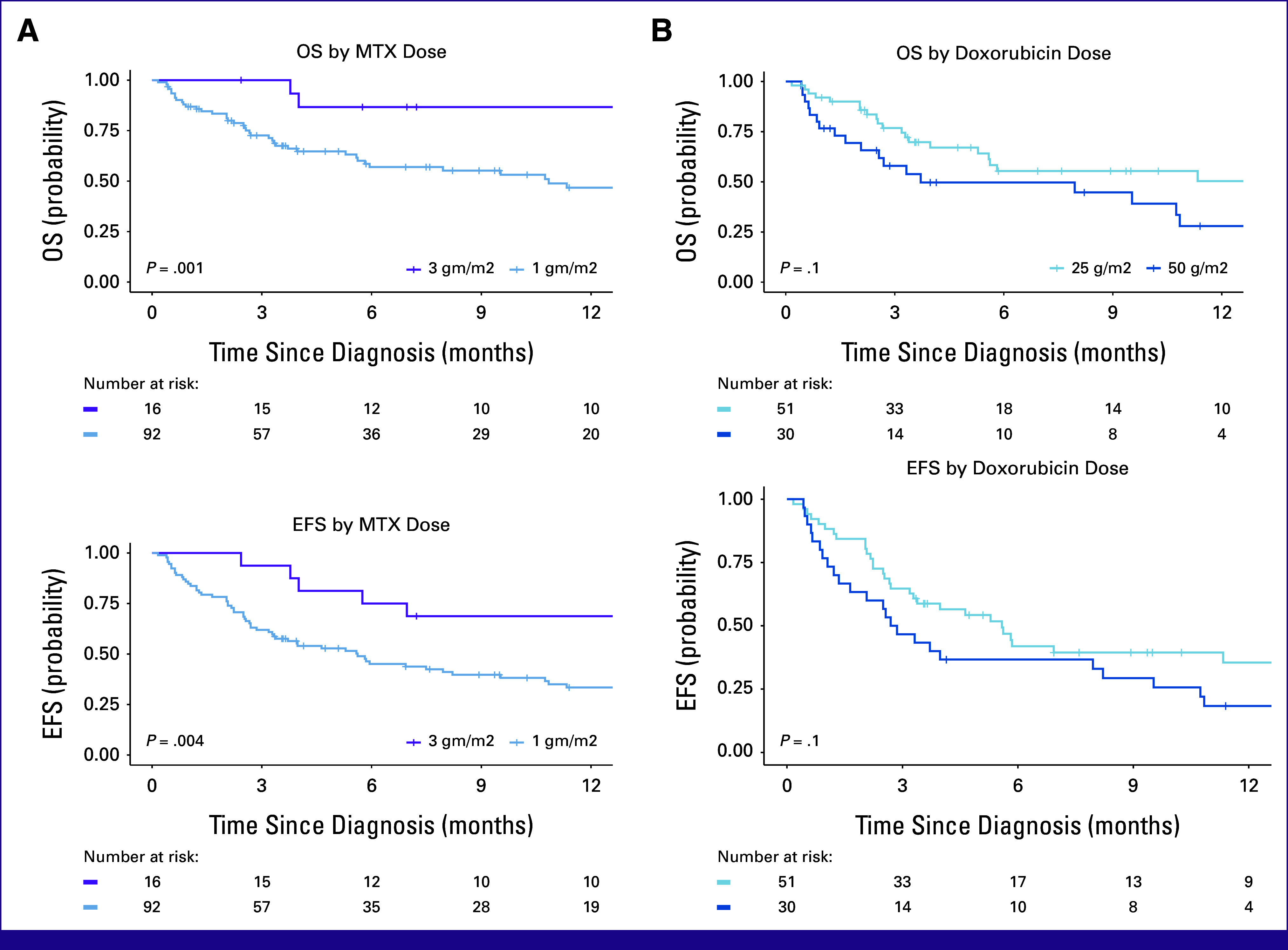
(A) Kaplan-Meier survival estimates for retrospective cohort of children with advanced MB-NHL by MTX. The 12-month OS among patients receiving MTX dosed at 3,000 mg/m^2^ was 87% (95% CI, 71 to 100) versus 47% (95% CI, 36 to 61) for 1,000 mg/m^2^ dose. The 12-month EFS for 3,000 mg/m^2^ was 69% (95% CI, 49 to 96) compared with 33% (95% CI, 24 to 46). (B) Kaplan-Meier survival estimates for retrospective cohort of children with advanced MB-NHL by anthracycline dose. All patients included received 1,000 mg/m^2^ of MTX, with either 25 mg/m^2^ or 50 mg/m^2^ of doxorubicin. The 12-month OS for patients receiving doxorubicin 25 mg/m^2^ was 49% (95% CI, 36 to 70) versus 28% (95% CI, 14 to 56), *P* = .14, for 50 mg/m^2^. EFS for 25 mg/m^2^ was 36% (95% CI, 23 to 54) versus 18% (95% CI, 8 to 40), *P* = .13, for 50 mg/m^2^. EFS, event-free survival; MB-NHL, mature B-cell non-Hodgkin lymphoma; MTX, methotrexate; OS, overall survival.

Of 108 patients, 60 (56%) completed their planned first-line therapy, 35 (32%) died before completion, and 13 (12%) abandoned treatment. With a median follow-up of 14 months (IQR, 9-25), 12-month EFS and OS were 39% (95% CI, 30 to 50) and 54% (95% CI, 44 to 66), respectively (Fig 2). The 12-month OS for 3,000 mg/m^2^ was 87% (95% CI, 71 to 100) compared with 47% (95% CI, 36 to 61; *P* = .001) for 1,000 mg/m^2^. MTX at 3,000 mg/m^2^ had EFS of 69% (95% CI, 49 to 96) compared with 33% (95% CI, 24 to 46; *P* = .004; Fig 3). Given small numbers in the 3,000 mg/m^2^ group, no subgroup analysis by stage was performed. There were 17 (16%) relapses: five CNS and 12 non-CNS relapses. There was no significant difference in OS or EFS between the two doses of doxorubicin—25 versus 50 mg/m^2^ (Fig 3).

#### Toxicity

Forty-nine deaths occurred. Twenty-eight (26%) died of treatment-related causes. Treatment deaths in the 1,000 mg/m^2^ group were 27 (28%) compared with the 3,000 mg/m^2^ group (n = 1/10, 10%; *P* = .45). Sixty-seven (62%) patients had at least 1 episode of febrile neutropenia. More febrile neutropenia was observed with the 3,000 mg/m^2^ (n = 12, 75%) than 1,000 mg/m^2^ (n = 55, 60%) dose, and 65% of episodes occurred in the first cycle.

#### Survival by Nutritional Status

**FIG A1:**
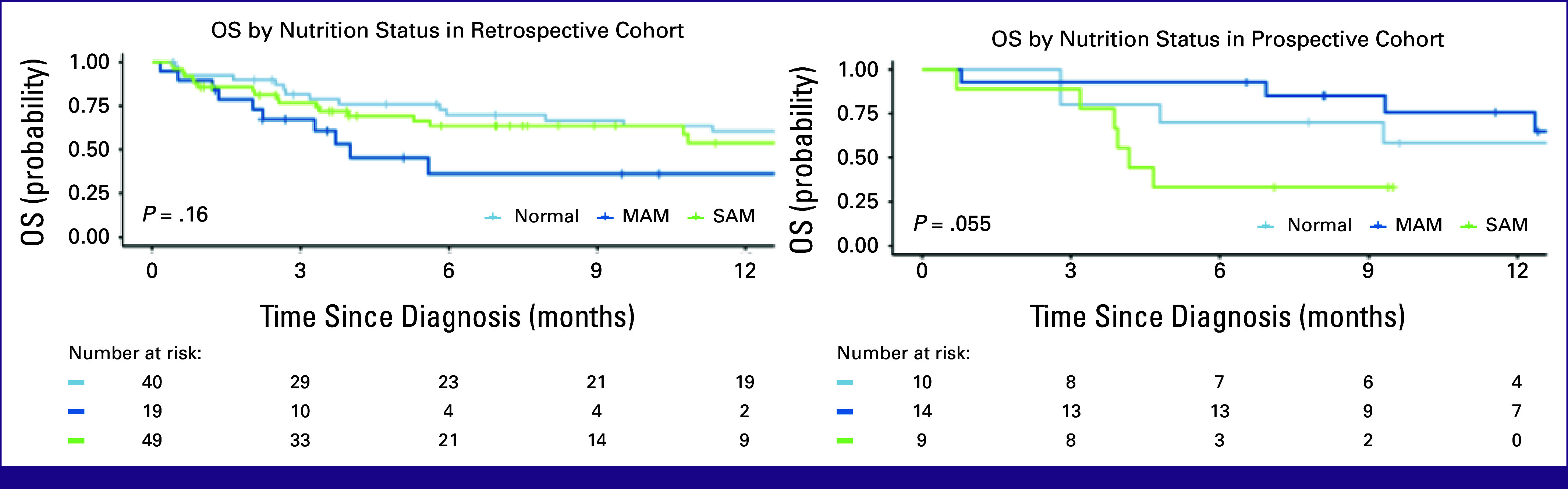
Kaplan-Meier survival estimates among children with advanced MB-NHL by nutritional status. The 12-month OS in the retrospective cohort with MAM was 36% (95% CI, 18 to 74) compared with those with SAM at 54% (95% CI, 39 to 74) and normal nutrition status at 60% (95% CI, 46 to 79; *P* = .2). The 12-month OS in the prospective cohort with MAM was 76% (95% CI, 55 to 100), those with SAM was 33% (95% CI, 13 to 84), and those with normal nutrition status was 58% (95% CI, 34 to 100; *P* = .055). MAM, moderate acute malnutrition; MB-NHL, mature B-cell non-Hodgkin lymphoma; OS, overall survival; SAM, severe acute malnutrition.

The 12-month OS with MAM was 36% (95% CI, 18 to 74) compared with 54% (95% CI, 39 to 74) in SAM or 60% (95% CI, 46 to 79; *P* = .2) for normal nutrition (Appendix Fig A1). Treatment-related mortality (TRM) accounted for 12 deaths with SAM (n = 49; 24%) and six (n = 19; 32%) with MAM (*P* = .8).

### Baseline Characteristics and Treatment in the Prospective Cohort

Between June 2022 and July 2023, 44 consecutive patients were diagnosed with advanced MB-NHL. Of these, 38 received HD-MTX, with six patients dying or abandoning treatment before getting a dose of HD-MTX. We included only these 38 patients in this analysis. Patients were further risk stratified as high-risk (HR) or VHR (Table 1). They were primarily male (n = 26, 68%) with a median age of 8 years (IQR, 6-11). Most (n = 20, 53%) were stage III, and 13 (34%) were stage IV. CNS and BM involvement were assessed in all patients. Four (10.5%) had CNS, and 11 (29%) had BM disease. Nine patients (24%) had SAM.

### Survival Outcomes in the Prospective Cohort

At the time of censoring, 23 (61%) had completed first-line therapy, eight (21%) died before completion, and seven (18%) abandoned. There were 110 HD-MTX administrations. Of these, 18 included rituximab. Sixteen (15%) of the cycles were delayed. For the non–rituximab-containing cycles (n = 92), there were 15 episodes (16%) of ≥grade 3 febrile neutropenia, two (2%) of ≥grade 3 mucositis, and eight (9%) of ≥grade 3 anemia. One patient developed grade 3 AKI, which resolved with hydration. In patients who received rituximab with HD-MTX (n = 18), there were four (22%) ≥grade 3 febrile neutropenia and one (6%) ≥grade 3 mucositis or anemia (Appendix Table A3).

There were 15 deaths—10 from disease progression, two from unknown or other causes, and three (8%) related to treatment with HD-MTX. Two treatment-related deaths were from neutropenic sepsis, and the third died of typhlitis.

Twenty-nine patients completed interim assessment at the end of the first consolidation, and 20 (69%) were in CR. Ten patients either relapsed or had progressed—five (50%) were stage IV.

#### Survival Estimates

**FIG 4:**
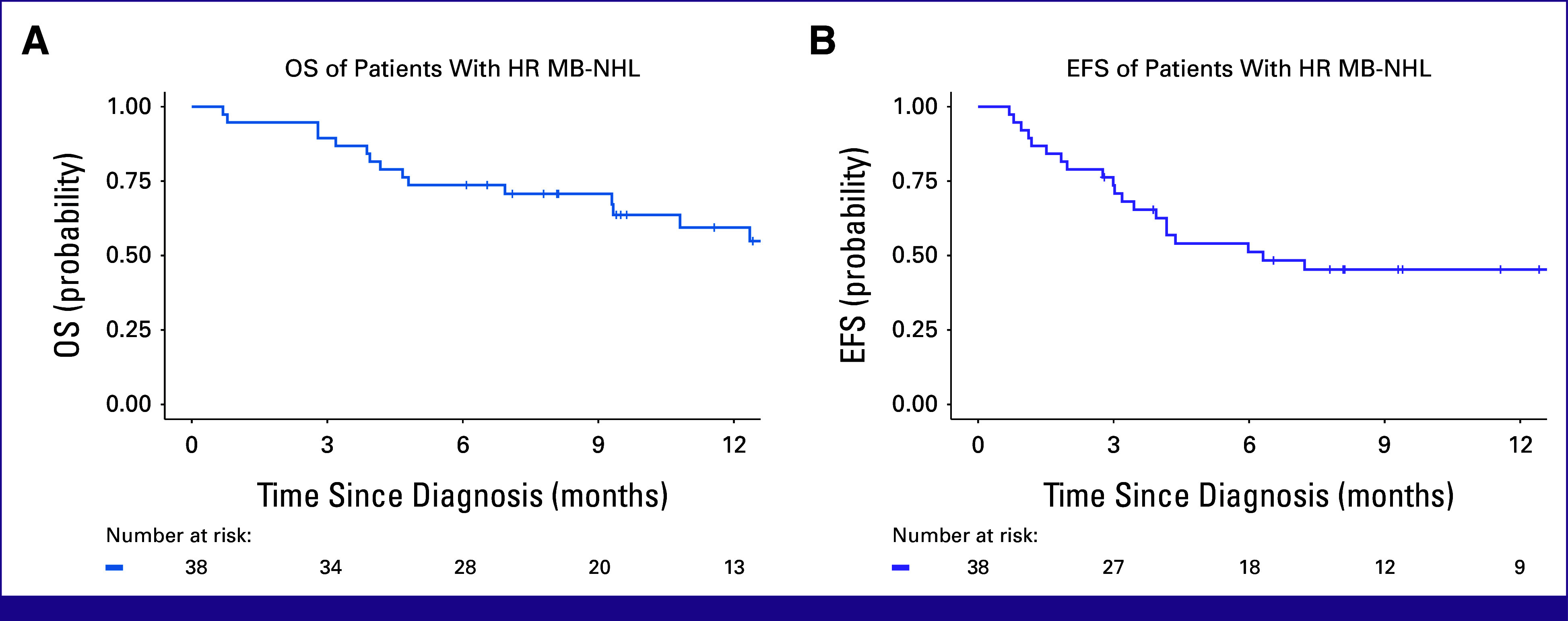
Kaplan-Meier 12-month OS and EFS estimates for a prospective cohort of children with advanced MB-NHL. The 12-month OS and EFS are 59% (95% CI, 44 to 79) and 46% (95% CI, 31 to 68), respectively. EFS, event-free survival; HR, high-risk; MB-NHL, mature B-cell non-Hodgkin lymphoma; OS, overall survival.

With a median follow-up of 14.5 months (IQR, 9.6-17.9), the 12-month EFS and OS were 45% (95% CI, 32 to 65) and 59% (95% CI, 45 to 79), respectively (Fig 4). The 12-month OS for those risk stratified as HR was 63% (95% CI, 47 to 84) versus 40% (95% CI, 14 to 100; *P* = .49) for VHR. HR had a 12-month EFS of 51% (95% CI, 35 to 75) versus 20% (95% CI, 3.5 to 100; *P* = .057) in VHR.

#### Survival by Nutritional Status

The 12-month OS was 76% (95% CI, 55 to 100) for MAM, 33% (95% CI, 13 to 84) for SAM, and 58% (95% CI, 34 to 100) with normal nutrition (*P* = .055; Appendix Fig A1). Two children with SAM (n = 9; 22%) and 1 child with MAM (n = 14; 7%) died of treatment-related causes. Most deaths in SAM occurred within 6 months of diagnosis.

## DISCUSSION

Our analysis demonstrates improved EFS with 3,000 mg/m^2^ of HD-MTX compared with 1,000 mg/m^2^. Given the small number of patients who received 3,000 mg/m^2^ in the retrospective cohort, we further evaluated for efficacy and clinical toxicity in a prospective cohort after establishing locally adapted supportive guidelines. Our findings show comparatively low rates of treatment-related deaths (8%) and toxicities in the absence of real-time serum MTX measurements. The rigorous supportive care measures likely explain this; however, we also note they received lower anthracycline doses.

While we did not observe similar outcomes with 3,000 mg/m^2^ of MTX in both cohorts (possibly because of small numbers and lower abandonment in the retrospective cohort), survival >50% in advanced MB-NHL is a significant improvement from prior reports in Malawi and supports a critical place for HD-MTX and good supportive care in low- and middle-income countries.^2,8^ Previous attempts at escalating treatment on this backbone in few sites in SSA resulted in unacceptable toxicity, with any potential gains from intensification, lost in the ensuing TRM.^16,28-30^ However, with rigorous surveillance and local adaptations to supportive care, our experience demonstrates feasibility of administering HD-MTX in low-resource settings. To mitigate toxicity, we established locally appropriate best practices for supportive care including longer bicarbonate-containing hydration in lieu of real-time MTX levels, adjustments to intravenous fluid with daily creatinine estimations, oral folinic administration by the nursing team, and granulocyte colony-stimulating factor in the first cycle given higher risk of myelosuppression (Appendix Table A1).

Vaishnavi et al^31^ evaluated administration of 100 cycles of HD-MTX to 53 children with leukemia and lymphoma in India, using empirical hydration and additional folinic acid doses without MTX-level monitoring. The results indicated HD-MTX could be safely given with manageable toxicities, particularly when combined with close monitoring of renal function and mucosal health. Although concerns remain on potential risk of renal failure and higher rates of relapse due to higher doses of folinic acid,^31,32^ sustained hydration with folinic acid rescue seems to effectively mitigate toxicity without compromising the drug's therapeutic efficacy.^33^

In the prospective cohort, half of the patients who relapsed were stage IV. Given higher treatment failure in this group, strategies to intensify treatment in the context of available support care resources are needed. Options include administering MTX doses ≥5,000 mg/m^2^ over 24 hours, adding high-dose cytarabine and rituximab. Our data only demonstrate feasibility of administering 3,000 mg/m^2^ of MTX and cannot be safely extrapolated to doses beyond that. Routine monitoring of MTX is still strongly recommended, particularly for higher doses.

Beyond HD-MTX, it is postulated that severity of myelosuppression and mucositis accompanying treatment on FAB/LMB backbone is likely a synergistic toxicity of both MTX and anthracyclines.^34^ Furthermore, evidence supporting effectiveness of anthracyclines in treating MB-NHL is inconsistent.^8-10,13^ We assessed survival in relation to anthracycline dose (25 *v* 50 mg/m^2^), and our results indicate it likely did not significantly affect outcome. This is similar to a Children's Cancer Group study in the 1980s, which reported comparable outcomes with or without daunomycin.^35^ Another possible interpretation could be the benefit of anthracyclines is not seen until other agents and supportive care have been optimized.

Acute malnutrition increases susceptibility to infections, complications, and treatment delays. Malnourished children may also experience altered drug metabolism, potentially affecting the safety and effectiveness of chemotherapy.^36-40^ Most children with SAM did not survive beyond 6 months in our prospective cohort. An intriguing finding emerged in the retrospective cohort regarding lower survival with MAM. We posit this discrepancy could be attributed to more intensive therapeutic nutritional interventions administered to patients with SAM versus MAM previously. With a stronger nutrition program, all patients with malnutrition in our unit now receive appropriate supplementation regardless of severity. Additionally, the combination of SAM with MTX ≥3,000 mg/m^2^ may result in more toxicity.

Our study was limited by inherent bias introduced by incomplete data collection and/or documentation, including missing CNS status in 50% of the retrospective cohort. We acknowledge these cohorts cannot be directly compared. Also, the small number of patients who received 3,000 mg versus 1,000 mg/m^2^ of MTX led to wide CIs. We attempted to address these limitations by studying the impact of HD-MTX prospectively, and despite the limitations, our experience and locally adapted supportive care guidelines for HD-MTX should provide valuable data for treatment centers in the region looking to escalate their treatment intensity.

We are prospectively evaluating the efficacy of a locally adapted risk-stratified treatment protocol for MB-NHL that incorporates rituximab, dose-reduced anthracycline, and 3,000 mg/m^2^ of HD-MTX for all patients with advanced MB-NHL. We anticipate this will generate more rigorous efficacy and safety data. Additionally, a concurrent study on pharmacokinetics of MTX in malnourished children is ongoing and should provide more insight on the impact of malnutrition on treatment outcomes.

HD-MTX at 3,000 mg/m^2^/cycle yielded promising outcomes in advanced MB-NHL and was not associated with significant clinical toxicity when resource-adapted rigorous, supportive care guidelines for MTX administration were established in the absence of routine levels. Stage IV disease had higher treatment failure and represents a subset who will benefit from further intensification beyond 3,000 mg/m^2^/cycle. The role of nutrition warrants further studies, as SAM seems to be associated with inferior outcomes.

## APPENDIX

**TABLE A1:** Supportive Care for Pediatric MB-NHL Protocol at KCH, Lilongwe, Malawi (June 2022-July 2023)

Supportive Care Routines for Patients on the Pediatric MB-NHL Protocol at KCH	Description
Routine monitoring	Fluid chart monitoring
Blood count monitoring	Start each cycle of therapy (exception, prephase and first cycle): ANC ≥1,000/µL and platelets ≥100,000/µL
	After cycle 1, routinely give GCSF 5 mcg/kg daily to all patients from day 7 and continue until ANC >1,000 postnadir
	Counts should be repeated at least 24 hours after the last dose of GCSF to confirm it is appropriate for chemotherapy
	Chemo should be started no <48 hours after the last dose of GCSF. If delayed count recovery by day 18, give GCSF 5 mcg/kg daily
MTX monitoring	IV Ringer lactate +25 mEq/L of NaHCO_3_ for 12 hours prehydration before MTX then continued for 96 hours after MTX infusion If urine pH <7 after prehydration or anytime during MTX infusion, increase NaHCO_3_ to 50 mEq/L
	Urine dipstick at least once every day for specific gravity and pH and aim at urine pH of ≥7 and specific gravity ≤1.010
	Folinic acid: start strictly 24 hours after MTX infusion start time, 15 mg/m^2^/dose orally 6 HRLY for 72 hours; 12 doses in total
	Nurses to directly observe the start time of folinic acid and every dose patient takes
	If serum creatinine rises by more than 25% of the pre-MTX infusion level, increase IV fluids to 200 mL/m^2^/h and increase folinic acid dose to 15 mg/m^2^/dose PO 3 HRLY if creatinine increase is >50%. Monitor creatinine levels daily until levels return to pre-MTX levels
	Discuss with the consultant the actual stop time for fluids, folinic acid, and NaHCO_3_; it may be extended
Laboratory tests	Creatinine, urea, sodium, and potassium on days 0, 2, and 3 routinely and as needed if abnormal
Prophylaxis	Co-trimoxazole prophylaxis at discharge for 1 week. No co-trimoxazole prophylaxis 24 hours before and after MTX

Abbreviations: ANC, absolute neutrophil count; GCSF, granulocyte colony-stimulating factor, HRLY, hourly; IV, intravenous; KCH, Kamuzu Central Hospital; MB-NHL, mature B-cell non-Hodgkin lymphoma; MTX, methotrexate; NaHCO_3_, sodium bicarbonate; PO, orally.

**TABLE A2:** Characteristics of Pediatric Patients With Advanced MB-NHL in the Retrospective Cohort

Patient Characteristic	1 g/m^2^ MTX (n = 98)	3 g/m^2^ (n = 10)	*P* ^a^
Age, years, median (IQR)	9 (6-10.3)	8 (6-10.5)	.7
Male sex, No. (%)	59 (60)	2 (20)	.17
Nutritional state at diagnosis, No. (%)			.06
Normal	35 (36)	5 (50)	
Moderate acute malnutrition	18 (18)	1 (10)	
Severe acute malnutrition	45 (46)	4 (40)	
Murphy stage at diagnosis, No. (%)			.27
Stage III	71 (72)	8 (80)	
Stage IV	27 (28)	2 (20)	
CSF positive at diagnosis, No. (%)	7 (7)	0	.08

NOTE. Comparison of MTX regimens (1,000 mg/m^2^ and 3,000 mg/m^2^).

Abbreviations: CSF, cerebrospinal fluid; MB-NHL, mature B-cell non-Hodgkin lymphoma; MTX, methotrexate.

^a^ Age between each cohort was assessed with the Welch *t*-test. Differences in proportions were assessed with the Pearson *c*^2^ test.

**TABLE A3:** Adverse Events in Rituximab and Non–Rituximab-Containing HD-MTX Cycles in the Prospective Cohort

Adverse Event	Rituximab-Containing HD-MTX Cycles (n = 17)	Non-Rituximab HD-MTX Cycles (n = 90)
Febrile neutropenia	Three episodes (18%)	14 episodes (16%)
Mucositis	One episode (6%)	One episode (1%)
Anemia	One episode (6%)	Five episodes (6%)
Sepsis	One episode (6%)	One episode (6%)

Abbreviation: HD-MTX, high-dose methotrexate.
